# Update on Pharmacological Activities, Security, and Pharmacokinetics of Rhein

**DOI:** 10.1155/2021/4582412

**Published:** 2021-08-17

**Authors:** Gang-Min Li, Jun-Ren Chen, Hui-Qiong Zhang, Xiao-Yu Cao, Chen Sun, Fu Peng, Yan-Peng Yin, Ziwei Lin, Lei Yu, Yan Chen, Yun-Li Tang, Xiao-Fang Xie, Cheng Peng

**Affiliations:** ^1^State Key Laboratory of Traditional Chinese Medicine Resources in Southwest China, Chengdu University of Traditional Chinese Medicine, Chengdu 610075, China; ^2^Department of Pharmacology, Key Laboratory of Drug-Targeting and Drug Delivery System of the Education Ministry, Sichuan Engineering Laboratory for Plant-Sourced Drug and Sichuan Research Center for Drug Precision Industrial Technology, West China School of Pharmacy, Sichuan University, Chengdu 610041, China; ^3^Guangxi University of Traditional Chinese Medicine, Nanning 530200, China

## Abstract

Rhein, belonging to anthraquinone compounds, is one of the main active components of rhubarb and *Polygonum multiflorum*. Rhein has a variety of pharmacological effects, such as cardiocerebral protective effect, hepatoprotective effect, nephroprotective effect, anti-inflammation effect, antitumor effect, antidiabetic effect, and others. The mechanism is interrelated and complex, referring to NF-*κ*B, PI3K/Akt/MAPK, p53, mitochondrial-mediated signaling pathway, oxidative stress signaling pathway, and so on. However, to some extent, its clinical application is limited by its poor water solubility and low bioavailability. Even more, rhein has potential liver and kidney toxicity. Therefore, in this paper, the pharmacological effects of rhein and its mechanism, pharmacokinetics, and safety studies were reviewed, in order to provide reference for the development and application of rhein.

## 1. Introduction

**Figure 1:**
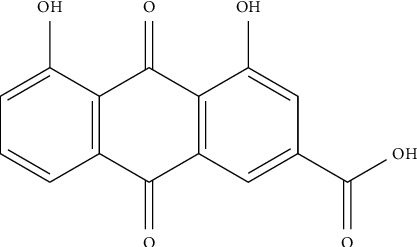
Chemical structure of rhein.

**Figure 2:**
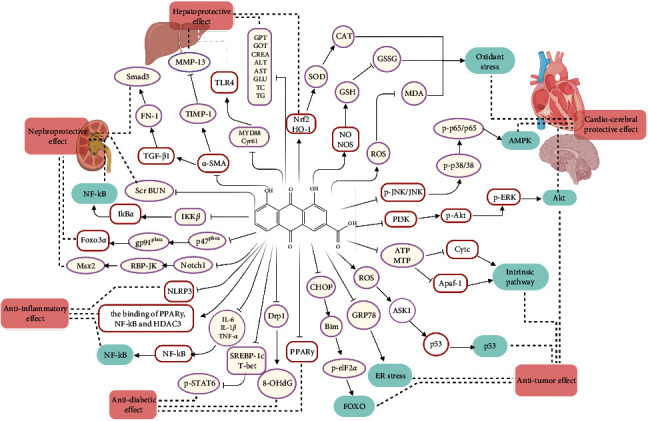
Molecular pathways involved in the pharmacological effects of rhein.

Rhein (4,5-dihydroxyanthraquinone-2-carboxylic acid, C_15_H_8_O_6_), with the molecular weight of 284.22, possessing an anthraquinone tricyclic aromatic structure, is insoluble in water, but soluble in alkaline solutions, such as pyridine and sodium bicarbonate aqueous solution. The structural formula is shown in Figure 1. Rhein comes from a wide range of sources, which could be isolated from a variety of traditional Chinese medicines, such as rhubarb (Dahuang), *Polygonum multiflorum* (Heshouwu), *Polygonum cuspidatum* (Huzhang), and cinnamon (Jue Mingzi) [1]. Modern studies have shown that rhein has cardiocerebral protective effect, hepatoprotective effect, nephroprotective effect, anti-inflammation effect, antitumor effect, antidiabetic effect, and so on. However, rhein has been reported to possess potential liver and kidney toxicity as well [2, 3]. Besides, due to the poor water solubility, the bioavailability of rhein is low, which greatly obstructs its clinical application [4, 5]. Therefore, clarifying its underlying dose-effect relationship and modifying the structure to improve the bioavailability and reduce the toxicity are of great importance for its utilization. Although a recent review has summarized the pharmacological effects of rhein and its derivatives, the pharmacological effects of rhein have not been fully elaborated, especially the relevant mechanisms. Besides, the research progress on the toxicity of rhein was not sorted out [6]. In this paper, the pharmacological properties and potential mechanisms, pharmacokinetics, and toxicity of rhein are comprehensively reviewed and analyzed, and the potential application prospects of rhein as a drug are prospected. The pharmacological effects and related mechanisms are summarized in Tables 1 and 2 and Figure 2.

## 2. Pharmacological Activities

### 2.1. Cardiocerebral Protective Effect

At present, there are as many as 330 million patients with cardiovascular disease (CVD) in China, including 13 million stroke patients, 11 million coronary heart disease patients, and 8.9 million heart failure patients [46]. Oxidative stress plays a vital role in the pathogenesis of various cardiovascular diseases, such as heart failure, myocardial ischemia-reperfusion injury, cardiomyopathy, and atherosclerosis [47]. Oxidative stress leads to excessive production of reactive oxygen species (ROS), and excessive ROS can cause severe damage to cardiomyocytes, which can give rise to a destruction of oxidation and antioxidant balance [48], and even cell death. Therefore, reducing oxidative stress or directly intervening cell apoptosis can provide potential molecular targets for cardiovascular diseases therapy [49].

Atherosclerosis (AS), as a chronic inflammatory disease that can lead to the deposition of oxidized lipoproteins and the formation of fatty plaques, accounts for 75% of the total mortality of CVD [50]. Studies have shown that vascular inflammation, especially at the level of endothelial cells, is responsible for the initiation, progression, and clinical complications of AS [51]. It has been reported that rhein can inhibit the damage of human umbilical vein endothelial cells (HUVECs) induced by hydrogen peroxide (H_2_O_2_). Specifically, rhein at different concentrations (2, 4, 8, and 16 *μ*mol/L) remarkably reduced the content of malondialdehyde (MDA) and lactate dehydrogenase (LDH) in H_2_O_2_-treated HUVECs, while increased the level of nitric oxide (NO) as well as the activity of nitrogen oxide synthase (NOS), superoxide dismutase (SOD), and glutathione peroxidase (GSH-PX). Furthermore, rhein pretreatment could downregulate the mRNA expression of Bid, caspase-3, caspase-8, and caspase-9, which was critical for blocking cell apoptosis. In conclusion, inhibiting excessive apoptosis of vascular endothelial cells induced by oxygen radical may be the key of rhein in AS prevention and treatment [7]. Moreover, 1 *μ*g/mL rhein can significantly reverse the damage of H9c2 cells induced by H_2_O_2_ through reducing the content of ROS, increasing the content of GSH-PX and CAT, and inhibiting the levels of proinflammatory factors IL-6, tumor necrosis factor-*α* (TNF-*α*), IL-1*β*, and IL-1*α*. Meanwhile, rhein activated the MAPK and NF-*κ*B signaling pathways, which thereby significantly reduced the protein expressions of p-JNK/JUK, p-P38/P38, and p-P65/P65 [8]. These phenomena demonstrated that alleviating oxidative stress and modulating apoptosis might be the potential molecular targets of rhein in CVD therapy.

Traumatic brain injury (TBI) is the main cause of long-term disability in children and young people worldwide, and oxidative stress mainly leads to the destruction of the blood-brain barrier (BBB) after TBI [52]. After TBI, upregulation of gp91^phox^ can significantly increase ROS by regulating macromolecular oxidation and redox signaling pathways. Subsequently, ROS activates ERK1/2-mediated degradation of MMP-9 and ZO-1 to regulate lipid and chromatin, which eventually leads to blood-brain barrier dysfunction. Recent studies illustrated that rhein (12 mg/kg) could downregulate the expression of MMP-9 mRNA and protein, while upregulating the expression of ZO-1 mRNA and protein in controlled cortical impingement (CCI) rats. Meanwhile, rhein could downregulate the expressions of GFAP and p-ERK *in vitro*, prevent the activation of gp91^phox^, and suppress the generation of ROS. Additionally, the cell injury index reflected by the downregulation of GFAP expression indicates that the neuroprotective effect of rhein is associated with the severity and prognosis of brain injury [9]. After intragastric administration, rhein could cross BBB. Rhein can increase the activity of SOD and catalase (CAT), the level of glutathione (GSH), and the ratio of GSH/GSSG and decrease the content of MDA and oxidized glutathione (GSSG) [10]. Therefore, these results indicated that rhein may be a potential therapeutic agent to protect BBB after TBI.

Ischemic stroke, with the increasing incidence rate, high morbidity, and mortality rate, is the third largest death disease in the world after coronary heart disease and cancer [53]. The survivors usually suffer from the sequelae of permanent brain injury, which leads to the decline of life quality and the increase of social burden [54]. Recent studies showed that rhein (50 and 100 mg/kg) treatment significantly improved the neurological function score (NFS) and infarct size of MCAO rats. The content of MDA was significantly increased, while the activities of SOD, GSH-PX, and CAT were significantly decreased. Compared with the ischemia/reperfusion (I/R) group, rhein suppressed the expression of Bcl-2 and enhanced the protein and mRNA expression of caspase-9 and caspase-3. These findings indicated that the protective effect of rhein is mediated by enhancing endogenous antioxidant defense and inhibiting oxidative stress pathway in ischemic rat brain, demonstrating that rhein might be a promising agent for treating ischemic stroke [11].

Myocardial infarction (MI) is a serious cardiovascular disease. Even though thrombolytic therapy and revascularization strategies have been applied to alleviate the occluded epicardial coronary arteries in MI patients [55], about 35% of patients still suffer from myocardial reperfusion damage after MI [56, 57], which can promote myocardial apoptosis and is closely related to heart failure [58, 59]. Previous studies have shown that Akt/GSK3 *β* pathways play a key role in heart protection; activation of Akt and GSK3*β* and the inactivation of caspase-1 are involved in ischemic preconditioning [60]. Pretreatment with 1 *μ*g/ml rhein for 1 h could significantly increase the viability of H9c2 cells and decrease its apoptosis as well as ROS production. In addition, rhein upregulated the phosphorylation of Akt (p-Akt) and GSK3*β* (p-GSK3*β*) and downregulated p-p38, which could be eliminated by LY294002 (an inhibitor of PI3K/Akt signaling) or GSK3*β*-siRNA [12]. In short, rhein can participate in myocardial protection by enhancing the oxidative defense system via upregulating Akt/GSK3 *β* pathways. Nowadays, mitochondrial quality control mechanisms are considered as molecular targets in cardiac ischemia-reperfusion injury, which provides new study direction for rhein's effect on MI [55–57].

### 2.2. Hepatoprotective Effect

A variety of reasons have been proved to be implicated in the pathogenesis of liver fibrosis, such as infections, viruses, drugs, genetics, and chemical poisons, which may lead to inflammation and necrosis of liver cells, stimulate the transformation of silent hepatic stellate cells (HSCs) into muscle fibroblasts (MFB), and release a large amount of *α*-SMA concurrently. Consequently, the synthesis and degradation of extracellular matrix (ECM) in liver are out of balance, leading to abnormal deposition of fibrous collagen tissue in the liver [61, 62]. The increase of *α*-SMA is a biomarker of HSC activation. Guo et al. used CCL_4_ to establish the liver fibrosis model in rats to investigate the hepatoprotective effect of rhein. They found that the structure of the liver lobules was disordered when exposed to CCL_4_, a large number of fibrous collagen tissues proliferated between the liver lobules, and the liver cord gaps were widened, accompanied by hepatocyte fatty degeneration and ballooning. However, these abnormal phenomena were reversed by rhein administration. Meanwhile, rhein observably reduced the expression of *α*-SMA, tissue inhibitor of metalloproteinase-1 (TIMP-1) protein, and mRNA in the rat liver and notably increased the expression level of matrix metalloproteinase-13 (MMP-13) [29]. To sum up, rhein can effectively reduce the protein and mRNA expression of *α*-SMA and TIMP-1 in liver fibrosis to delay the progress of liver fibrosis.

Remarkably, rhein can alleviate the hepatotoxicity induced by drugs such as acetaminophen (APAP) and methotrexate (MTX) [63]. Rhein at the doses of 40, 20, and 10 mg/kg could attenuate the hepatotoxicity in APAP-treated rats in a dose-dependent manner, manifesting as significantly reducing the levels of alanine aminotransferase (ALT) and aspartate aminotransferase (AST), and ROS in serum, and markedly decreasing the content of NO, MDA, and GSH. Furthermore, rhein treatment significantly attenuated liver cell necrosis and steatosis [30]. MTX is an antimetabolic and antitumor drug, which acts as a basic drug for treating rheumatoid arthritis and cancer in clinic. However, MTX possesses severe hepatotoxicity with a narrow treatment range [64]. Rhein (20, 50, and 100 mg/kg) can significantly lower the serum ALT and AST levels in rats caused by MTX. In addition, rhein also upregulated the mRNA and protein levels of nuclear factor-erythroid 2-related factor 2 (Nrf2), Bcl-2, heme oxygenase-1 (HO-1), and the catalytic subunit of glutamate-cysteine ligase (GCLC) in rats, downregulated the mRNA and protein levels of Bax. Besides, rhein also reduced the protein expression of NF-*κ*B, TNF-*α*, and caspase-3, improved the survival rate of LO2 cells, and reduced the rate of apoptosis [31]. These results have illustrated that rhein is capable of reducing drug-induced liver injury through activating the Nrf2-HO-1 antioxidant pathway.

Nonalcoholic fatty liver disease (NAFLD) is a clinical syndrome characterized by steatosis and fat storage of hepatic parenchymal cells without a history of excessive alcohol consumption, which can be divided into nonalcoholic fatty liver disease (NAFL) and nonalcoholic steatohepatitis (NASH). Recent studies have shown that oxidative stress and inflammatory response play a significant role in the occurrence and development of NAFLD [65]. Abnormal activation of the TLR4/NF-*κ*B signaling pathway may be involved in the occurrence of NAFLD [66]. TLR4 is a toll-like receptor with the strongest correlation with NAFLD. TLR4 recognizes the pathogenic model molecule and activates NF-*κ*B through the downstream MYD88-dependent pathway, which is the most critical transcription factor in the inflammatory response [67]. Furthermore, the excessive expression of NF-*κ*B is able to activate the inflammatory response and leads to severe liver damage. 100 mg/kg rhein significantly reduced the levels of ALT, AST, GLU, TC, TG, and MDA in the serum of NAFLD rats, while significantly increased the levels of SOD and GSH-PX. In addition, rhein downregulated the expression of TLR4, MYD88, and Cyr61 in rat liver tissues [32]. These phenomena demonstrated that rhein could alleviate both liver disfunction and liver structure in NAFLD rats by inhibiting the TLR4 receptor pathway.

### 2.3. Nephroprotective Effect

Renal interstitial fibrosis, pathologically characterized by fibroblast proliferation and excessive deposition of extracellular matrix in the renal interstitium, is a common pathway for the progressive development of chronic kidney disease (CKD) and the main cause of chronic renal failure [68, 69]. He et al. found that rhein (150 mg/kg) significantly improved renal interstitial fibrosis and reduced the expression of *α*-SMA as well as the deposition of fibronectin (FN) in a mouse model of renal interstitial fibrosis induced by unilateral ureteral obstruction (UUO). In addition, rhein also inhibited the expression of transforming growth factor-*β*1 (TGF-*β*1) and its type I receptor. Further studies showed that rhein also reduced the expression of *α*-SMA and FN in renal interstitial fibroblasts (NRK-49F) *in vitro* [33]. Klotho is a renal specific antifibrosis protein, which is essential for maintaining renal homeostasis. Rhein administrated (pretreatment for 1 day and posttreatment for 3 days) at 120 mg/kg effectively reversed the abnormal expression of DNA methyltransferase 1 and 3a in UUO mice and maintained the secretion (sKL) and membrane Klotho (mKL) levels [34], leading to alleviate fibrosis. Moreover, rhein significantly reversed the adenine (Ade)-induced thinning, disorder, increased lacunar, and decreased bone density in the trabecular bone of mouse femurs and restored the abnormal expression of E-cadherin, *α*-SMA, *β*-catenin, and phosphorylated Smad3 (p-Smad3). Besides, rhein also corrected the hypermethylation induction of DNA methyltransferase DNMT1/DNMT3a and Klotho promoter [35]. However, Klotho Gene knockout by siRNA largely eliminated the antirenal fibrosis effect of rhein, which indicated that the point in rhein's renal protective effect was reversing Klotho deficiency. Meanwhile, rhein can alleviate renal fibrosis and autophagy in rats with renal tubule injury and lower the levels of collagen I, FN, and LC3 II. *In vitro*, 10 *µ*g/mL rhein inhibited the autophagy of NRK-52E cells induced by Ade by regulating AMPK-dependent mTOR, ERK, p38 MAPK, and Akt-dependent signaling pathways [36]. In the 5/6 nephrectomy-induced rat CKD model, rhein (50, 100, and 150 mg/kg) significantly reduced the levels of serum creatinine (Scr) and blood urea nitrogen (BUN), the content of ROS, and the expression of NADPH oxidase subunit p47^phox^ and gp91^phox^. Furthermore, the effect of rhein on H_2_O_2-_treated HK-2 cells is consistent with that of the above animal experiments, simultaneously increased FOXO3*α* expression. However, SIRT3 knockout abolished the antifibrotic effect of rhein [37], which implied that the renal protection of rhein could be attributed to activate the SIRT3/FOXO3*α* signaling pathway.

Hyperuricemia is closely related to the overproduction and insufficient excretion of uric acid, which has always been considered as a pivotal risk factor for kidney disease. When the human body is unable to participate in the normal physiological process of purine degradation and elimination, as well as the normal excretion of uric acid in the kidney, the concentration of uric acid in the blood will increase nonphysiologically and abnormally [70]. Once the local or serum uric acid concentration is beyond the solubility of uric acid, the urate crystal forms locally and does harm to the body. Urate crystals cause uric acid kidney stones, which in turn stimulate strong inflammatory responses [71]. Therefore, preventing hyperuricemia and alleviating inflammation are necessary for the treatment of uric acid nephropathy. Studies reported that rhein significantly inhibited the levels of serum uric acid (Sur), Scr, BUN, xanthine oxidase (XOD) activity, and creatinine clearance rate (CCr) in adenine and ethambutol induced uric acid nephropathy (UAN) mice. Meanwhile, it also significantly reduced inflammatory factors (TNF-*α*, prostaglandin E2 (PGE2), IL-1*β*, and TGF-*β* 1. Additionally, Rhein can distinctly reduce Sur level by inhibiting the activity of XOD and increasing the excretion of uric acid, thus preventing hyperuricemia in mice [38].

Acute kidney injury (AKI) is one of the main causes of kidney diseases. The death of renal tubular cells and the subsequent proinflammatory response are considered to be the dominating features of various forms of AKI [72, 73]. AKI is a clinical syndrome characterized by sudden decrease of renal function and accumulation of metabolic waste [74], which is a common organ injury in the intensive care unit (ICU). Sepsis is the main cause of AKI, accounting for 50% of all cases [75]. Studies have shown that rhein can improve renal function and alleviate renal injury in septic mice. Specifically, rhein significantly reduced the concentrations of BUN, Scr, TNF-*α*, and IL-1*β* in two different experimental sepsis models in mice induced by lipopolysaccharide (LPS) and cecal ligation and puncture (CLP), respectively. In addition, rhein obviously suppressed the infiltration of circulating leukocytes and enhanced the phagocytic activity of macrophages partially damaged at 12 h after CLP. Further study showed that rhein enhanced the viability of HK-2 cells stimulated by LPS and inhibited the release of MCP-1 and IL-8. Notably, rhein even downregulated the expression of phosphorylated NF-*κ*B p65, I*κ*B*α*, and IKK*β* stimulated by LPS both *in vivo* and *in vitro* [39].

Diabetic nephropathy (DKD) refers to the renal damage caused by chronic hyperglycemia, which is one of the most serious microvascular complications of diabetes, and also the primary cause of chronic kidney disease worldwide [75, 76]. Wang et al. used high-fat diet and streptozotocin to establish the obese diabetic nephropathy rat model to investigate the role of rhein in DKD. After treatment with rhein (100 mg/kg) for 8 weeks, the content of MDA in kidney tissue was reduced and the activity of SOD was significantly increased. Meanwhile, rhein reduced the excretion of urinary protein, the blood sugar, and body weight of the rats. In short, rhein can prevent diabetic nephropathy and the underlying mechanism might be associated with its antioxidant activity [40]. Besides, aortic calcification may occur in the progression of diabetic nephropathy. Duan et al. found that rhein improved the calcification of abdominal aorta in diabetic nephropathy through activating Notch1-RBP-JK/Msx2 pathway, alleviating the levels of serum calcium and serum phosphorus [41].

### 2.4. Anti-Inflammatory Effect

Long-term use of nonsteroidal anti-inflammatory drugs and cyclooxygenase inhibitors may cause a series of adverse events such as gastrointestinal ulcers, bleeding, and liver and kidney dysfunction. Therefore, it is urgent to develop other anti-inflammatory drugs. The I*κ*B kinase P (IKK*β*)/nuclear transcription factor (NF-*κ*B) pathway is known as the most promising target for anti-inflammatory drug in recent studies. Rhein is the active metabolite of diacerein, which exerts anti-inflammatory activity mainly through the IKK*β*/NF-*κ*B pathway without gastrointestinal damage [77].

The NF-*κ*B signaling pathway plays a key role in the process of inflammation as its activation promotes the release of proinflammatory mediators, such as inducible nitric oxide synthase (iNOS) and TNF-*α*. Gao et al. found that rhein alleviated the inflammatory response of RAW264.7 cells stimulated by LPS. Rhein inhibited the activation of NF-*κ*B and the downstream signal iNOS, while enhanced the release of IL-6, TNF-*α*, and IL-1 *β*. In addition, rhein enhanced the activity of caspase-1 by inhibiting intracellular (in situ) IKK*β*, which in turn increased the release of IL-1 *β* and high-mobility-group box 1. Unexpectedly, rhein visibly enhanced TNF-*α* secretion and phagocytosis in macrophages due to IKK*β* inhibition with or without LPS [13]. Furthermore, Wen et al. found that rhein evidently improved the survival rate of RAW264.7 cells. Rhein inhibited the protein and mRNA of NF-*κ*B p65, but this effect was eliminated by GW9662 (PPAR*γ* Inhibitors). And rosiglitazone (PPAR*γ* activator) promoted the inhibition of inflammatory factors and NF-*κ*B expression by rhein. Interestingly, rhein enhanced the binding of PPAR*γ*, NF-*κ*B, and histone deacetylase 3 (HDAC3). These findings indicated that rhein could exert its anti-inflammatory function by regulating the PPAR*γ*-NF-*κ*B-HDAC3 axis [14]. Besides, Hui et al. found that rhein distinctly reduced the abnormal migration of zebrafish immune cells induced by tail cutting as well as the expression of NLRP3 in RAW264.7 cells induced by LPS and ATP [15].

Sepsis refers to the systemic inflammatory response syndrome caused by infection, usually caused by the massive release of various toxins into the blood and tissues by pathogenic microorganisms after a severe infection. Intestinal barrier dysfunction may cause ischemia and hypoxia, further aggravating sepsis infection [78]. Moreover, toll-like receptor 4 (TLR4) plays a crucial role in the pathogenesis of inflammatory diseases, and the activation of TLR4 during sepsis may be related to the stimulation of lipopolysaccharide (LPS) endotoxin [79]. 100 mg/kg rhein or 0.3 mg/kg TAK-242 (TLR4 receptor inhibitor) could lengthen the colon and reduce colon damage. And rhein distinctly reduced the expression of IL-1*β*, IL-6, IL-8, and TNF-*α* in plasma and colon tissue induced by LPS. And rhein decreased the expression of TLR4 and inhibited the phosphorylation of NF-*κ*B. Therefore, rhein may alleviate LPS-induced intestinal inflammation through the TLR4 pathway [16].

### 2.5. Antitumor Effect

Multiple studies have proved that rhein has a broad-spectrum antitumor effect [80, 81]. Rhein mainly exerts the antitumor activity by inhibiting tumor cell proliferation, inducing tumor cell apoptosis, inhibiting tumor cell invasion and metastasis, inhibiting tumor angiogenesis, and regulating the level of intracellular oxidase. The underlying signaling pathways mainly involve the PI3K/Akt signaling pathway, p53 signaling pathway, mitochondria-mediated signaling pathway, FOXO signaling pathway, and MAPK signaling pathway [82].

Siu et al. found that the metabolic rate of mitochondria and cell proliferation of tumor cells (PANC-1, LTC-14, SW480, and SW620 cells) decreased gradually with increasing concentrations of rhein. Rhein (20 *μ*M) dramatically inhibited the upregulation of TGF-*β*1-induced oncogenic mediators MMP2, MMP-9, and EMT molecule CDH2, as well as the augment of Akt phosphorylation [17].

Breast cancer is one of the most common cancers in women and the second leading cause of cancer death. Hypoxia is the characteristic of breast cancer and other solid tumors, whose degree is related to treatment resistance and poor prognosis [83]. Fernand et al. found that the IC_50_ values of rhein to MCF-7 cells and MDA-MB-435S cells were 81.3 or 71.3 *μ*M and 52.1 or 127.3 *μ*M, respectively, when cultured under normal conditions or hypoxic conditions for 48 h. In addition, MCF-7 cells were arrested in the S phase, and MDA-MB-435S cells were accumulated in the G2/M phase. Rhein promoted the degradation NF-*κ*B, COX-2, and HER2 by inhibiting the activity of HSP90*α*. Furthermore, rhein inhibited phosphatidylinositol 3-kinase (PI3K), phosphorylated Akt (p-Akt), and phosphorylated extracellular signal-regulated kinase (p-ERK). In conclusion, rhein promoted NF-*κ*B degradation by inhibiting the PI3K/Akt/ERK pathway, thereby inhibiting tumor growth [18]. The research shows that NF-*κ*B is a key factor in the initiation and development of cancer, which promotes the expression of genes associated with tumor cell proliferation, inflammation, and angiogenesis [84]. Additionally, human breast cancer cells that overexpress HER2/neu are more aggressive and resistant to chemotherapy, which can result in a poor prognosis in patients. Therefore, Chang et al. studied the antiproliferation effects of 7 anthraquinone derivatives on human breast cancer cells and found that only rhein had antiproliferation and apoptosis effects on both MCF-7 (MCF-7/HER2) cells overexpressing HER2 and the control vector MCF-7 (MCF-7/VEC) cells. Rhein promoted the protein expression of caspase-9, making S phase arrest of MCF-7/HER2 cells and increase of G1 phase of MCF-7/VEC cells. In addition, rhein significantly increased the release of ROS and the expression of ASK1 and p53. In conclusion, rhein has antiproliferative activity on two types of breast cancer cells HER2 overexpression or HER2-basal expression and may induce apoptosis by activating ROS-mediated p53 signaling pathways [19]. However, the latest study indicates that NF-*κ*B is a tumor promoter and suppressor and plays a vital role in tumor promotion and suppression [84]. ROS easily interacts with DNA and other biological molecules, giving rise to the DNA damage and mutations in normal cells. ROS activated NF-*κ*B to support cancer cell survival by increasing the levels of antioxidants to escape cancer cell death [85].

Li et al. found that rhein changed the morphology and inhibited proliferation of SGC-7901, with cell viability downregulation by 15%. Rhein also induced apoptosis of SGC-7901 cells with the maximum apoptosis rate 43.5% at the concentration of 300 *μ*M, the mechanism referred to reducing the protein levels of Bcl-xL and procaspase-3, increasing the ratio of Bax/Bcl-2, and markedly increasing the expression of cytochrome c and apoptotic protease activator 1 (Apaf-1), which acts as key target for regulating mitochondrial pathway-mediated apoptosis [20].

Du et al. found that rhein induced mitochondrial swelling and Ca^2+^ leakage in HepG2 cells, but completely blocked by cyclosporine A (CsA), a specific blocker of mitochondrial permeability transformation. Rhein signally decreased the ATP content in HepG2 cells, leading to the loss of mitochondrial transmembrane potential (MTP), the release of Cyt c, and the activation of caspase-3. However, the toxic effects of rhein on HepG2 cells were all attenuated by CsA. Even more, CsA can significantly inhibit the apoptosis of HepG2 cells induced by rhein [21]. Rhein inhibited the activity of MCF-7 cells and HepG2 cells with IC_50_ values of 37.8 and 34.5 *μ*M, respectively, and induced atypical unfolded protein responses in MCF-7 cells and liver cancer HepG2 cells. Rhein suppressed the expression of CHOP and Bim, phosphorylation of eIF2a, and caspase cleavage. Meanwhile, rhein inhibited the expression of GRP78 and the splicing of X-box binding protein 1 induced by endoplasmic reticulum stress. Besides, rhein restrained p-Akt and stimulates FOXO transactivation activity. However, the knockout of FOXO3a or Bim eliminated the caspase lysis and apoptosis induced by rhein. In general, FOXO3a-mediated Bim upregulation may be a potential key mechanism of rhein-induced cancer cell apoptosis [22].

Heo et al. found that 5 *μ*M rhein promoted the expression of CD11b and CD14 in acute promyelocytic leukemia (APL) and enhanced the production and phagocytosis of ROS. Meanwhile, rhein motivated the expression of CCR1 and CCR2 to enhance ATRA-induced differentiation of macrophages in NB4 cells. In addition, rhein lowered MMP, but activated the expression of caspase-3, and activated ERK by promoting CD11b expression. Consequently, rhein differentiation therapy may contribute to the maturation of CD11b + macrophages and is beneficial for the treatment of APL [23].

DNA methylating agents can continuously damage nucleic acids in living cells, such as SN1 and SN2 agents, which causes DNA strand breaks, and methyl methanesulfonate salt (MMS), which produces n1-methyladenine (m1A) and n3-methylcytosine (m3C) damage in single-stranded DNA (ssDNA). The accumulation of m1A and m3C damage in single-stranded DNA (ssDNA) was usually very cytotoxic and even leaded to cell death. So far, AlkB repair seemed be the main natural defense mechanism, with the ability to restore the typical base structure in the body. Li et al. found that rhein had a strong inhibitory effect on E. *coli* AlkB *in vitro* in a dose-dependent manner. Experiments had shown that rhein can directly bind to AlkB in *E. coli* and can inhibit the human homologues ALKBH2 and ALKBH3 to sensitize U87 cells to MMS. The combination of rhein or MMS or rhein and MMS cannot change the cell abundance of H3K9me3, which showed that rhein had no inhibitory effect on JMJD2A or JMJD2E (two iron (II) and 2OG-dependent histone demethylases) at the tested concentration, proving that rhein had an effect on MMS alkylation damage sensitization due to ALKBH2 and ALKBH3 inhibition. In conclusion, rhein inhibited AlkB repair enzymes (AlkB, ALKBH2, and ALKBH3) *in vitro* and reduced cell resistance to MMS, and it was speculated that ALKBH2 and ALKBH3 enzymes may be effective pharmacological targets for overcoming tumor resistance to methylated anticancer drugs [86].

### 2.6. Antidiabetic Effect

Studies have shown that rhein can prevent and treat metabolic diseases such as obesity [25], diabetes [27], hyperlipidemia, and nonalcoholic fatty liver [32] disease. Rhein regulates glucose and lipid metabolism by inhibiting the absorption of cholesterol, reducing lipoprotein synthesis and blood glucose, regulating blood lipids, and improving insulin resistance [87].

Wang et al. found that rhein significantly increased energy expenditure in high-fat diet-induced obesity (DIO) mice, reduced body weight, improved insulin resistance, and lowered circulating cholesterol levels without affecting food intake. In addition, rhein also normalized serum ALT level, reduced liver triglyceride (TG) level in liver tissue, and finally reversed liver steatosis. Meanwhile, rhein markedly inhibited the expression of lipase sterol regulatory element binding protein 1c (SREBP-1c) and its target genes in DIO mice but did not reduce the weight of liver X receptor (LXR) knockout mice. Additionally, rhein inhibited the expression of T-box (T-bet) in T cells and enhanced the phosphorylation of signal transducer and activator of transcription 6 (STAT6), which thereby enhanced GATA binding protein 3 (GATA-3) and changed Th1/Th2 reaction [26]. On the other hand, rhein reversed the proinflammatory aggregation and microbiota changes in mouse large intestinal macrophages induced by the HF diet, including a decrease in *Bacteroides-Prevotella* spp. and *Desulfovibrio* spp. DNA and an increase in *Bifidobacterium* and *Lactobacillus* DNA. Rhein dramatically reduced the concentration of plasma lipopolysaccharide in the colon and the aggregation of M1 macrophages. Simultaneously, rhein improved BDNF levels in the peripheral cortex in obese mice and suppressed inflammation in the perinasal cortex [26]. Further research demonstrated that rhein reduced fat mass in DIO mice and the quantities of obese white as well as brown fat cells and decreased the levels of cholesterol, low-density lipoprotein cholesterol, and fasting blood glucose in serum. Even more, rhein inhibited the signal transduction of peroxisome proliferator-activated receptor *γ* (PPAR*γ*) and the expression of its target genes, indicating that rhein may be a PPAR*γ* antagonist [27].

Yu et al. found that rhein (100 mg/kg) reduced the fat weight of db/db mice, which emodin showed on effect [27]. Huang et al. found that rhein memorably increased the survival rate of pancreatic cancer B cells induced by ROS inhibitor (hg) and significantly inhibited B cell apoptosis. Interestingly, rhein is mainly located in the mitochondria of b cells. Rhein can maintain the ultrastructure of mitochondria by eliminating the abnormal expression of mitochondrial fission protein dynein related protein 1 (dynein related protein 1, Drp1) caused by hyperglycemia. Meanwhile, rhein markedly inhibited mitochondrial Drp1 levels and reduced the early blood glucose levels of db/db mice, the expression of NF-*κ*B, and the content of 8-hydroxydeoxyguanosine (8-OHdG), while signally enhanced mouse insulin staining [28].

### 2.7. Antimicrobial Effect

Studies showed that rhein had antibacterial effects on *Staphylococcus aureus* [88], *Helicobacter pylori*, and acne pathogens, with minimum inhibitory concentration (MIC) values of 15.625 *μ*g/mL, 50 *μ*g/mL, and 31.25 *μ*g/mL, respectively [89, 90]. Furthermore, compared with other ingredients isolated from Kampos, Liao et al. reported that rhein has the strongest antibacterial effect on *Porphyromonas gingivalis,* with an MIC value of 0.78 mg/mL [91]. Azelmat et al. measured the MIC value of rhein by broth microdilution assay and found that the MIC value of rhein was 2.5 mg/mL. The results of quantitative RT-PCR showed that rhein observably reduced the expressions of the host colonization-related genes fim A, hag A, and hag B. In brief, these phenomena indicated that the antibacterial activity of rhein mainly mediated by impairing the pathogenicity of *P. gingivalis* through reducing transcription of gene coding for important virulence factors [92].

### 2.8. Others

Pancreatic fibrosis is an important histopathological feature of chronic pancreatitis (CP) and pancreatic ductal adenocarcinoma. Pancreatic stellate cells (PSCs) play a vital point in the fibrogenesis of CP. TGF-*β*1 is a key regulator of extracellular matrix production and PSC activation [93]. Siu et al. found that rhein significantly reduced *α*-SMA and TGF-*β* in CP mice, thereby inhibiting the activation of PSCs, which is the key to fibrogenesis. In addition, the large amount of extracellular matrix FN1 and type I collagen (COLI-*α*1) deposition in the exocrine parenchyma was correspondingly reduced. In addition, rhein also inhibited the upregulation of SHH signal [94]. Interestingly, rhein can also prevent and treat pulmonary fibrosis in rats. Qu et al. replicated the rat model of pulmonary fibrosis by injecting bleomycin (5 mg/kg), and they found that rhein not only markedly reduced the degree of alveolitis and pulmonary fibrosis in rats but also significantly suppressed the coefficient of lungs and the content of hydroxyproline in lung tissues. In addition, rhein restrained the mRNA and protein expression of miR-21 and TGF-*β*1 in lung tissues, while increased the expression of Smad7 [95].

Another study showed that rhein has antiallergic activity. Rhein at 5 mg/kg displayed effective inhibition in mast cell degranulation. Besides, rhein significantly inhibited LOX enzyme activity, with IC_50_ value of 3.9 g/mL, suggesting that rhein can exhibit antiallergic activity by stabilizing or inhibiting LOX activity in mast cells, which indicates that rhein may be a potential therapeutic agent to treat allergic diseases [96].

## 3. Toxicological Effects

In the past decade, increasing adverse reactions caused by large dose or long-term administration of Heshouwu (*Polygonum multiflorum*) or its preparations have been reported and attracted widespread attention [97–99]. Therefore, selecting and determining the toxicity of its ingredients is crucial at present. In the acute toxicity test, the LD_50_ of rhein gavage mice was 2.1856∼4.6143 g/kg [100]. Besides, a large number of studies have shown that rhein, belonging to rhubarb extracted from Dahuang and Heshouwu, has potential liver and kidney toxicity as well [101].

### 3.1. Hepatotoxicity

Rhein could inhibit the proliferation of HepaRG cells with the IC_50_ value of 77.97 *μ*M, and it significantly decreased the mitochondrial membrane potential (MMP), arrested the cells in S phase, and caused a significant increase in apoptosis-related proteins such as Fas, p53, Bax, caspase-3, caspase-8, caspase-9, and others. These data showed that rhein could induce hepatotoxicity by inhibiting the Fas death pathway [42]. In addition, the IC_50_ offset value was 1.989 for NADPH preincubated rhein versus non-preincubated rhein, inferring that rhein can significantly reduce the activity of CYP2C19. Besides, reduced GSH trapping experiments demonstrated that CYP2C19 metabolizes rhein into an epoxy compound, which bound to intracellular mitochondria, resulting in the excessive production of ROS and increase of AST and LDH. These data showed that rhein impaired liver function and mitochondrial respiratory chain dysfunction, leading to cell death. However, CYP2C19 inhibitors can restore mitochondrial membrane potential and AST levels, which further showed that CYP2C19 could mediate the hepatotoxicity of rhein [43]. Furthermore, Yang et al. applied different concentrations (0, 175, and 375 mg/kg) of rhein to aged and immature mouse induced by D-galactose. The results illustrated that the mortality rate of elderly mice treated with 375 mg/kg rhein was 55.5%. And rhein increased the generation of ROS and MDA, reduced the levels of SOD and MMP, and aggravated mitochondrial swelling. In summary, the above results indicated that rhein induced oxidative stress and led to mitochondrial dysfunction and liver toxicity [100].

### 3.2. Nephrotoxicity

Sun et al. found that rhein could exert cytotoxic effect and apoptosis-inducing effect through the Fas signal pathway in HK-2 cells. Rhein (25, 50, and 100 *μ*mol/L) increased the release of lactate dehydrogenase (LDH) and elevated the expression of transmembrane protein death factor (Fas), death factor receptor (FasL), Fas-associated with death domain protein (FADD), caspase-3, caspase-8, and cytoplasmic cytochrome C (Cyt c), while downregulated caspase-8 prototype expression, thereby inhibiting HK-2 cell viability [44]. In addition, Hu et al. also found that long-term administration of rhein in large dose had certain toxicity to the kidney of mice. With the treatment of rhein (0.175 and 0.35 g/kg) for 60 days, the weight of mice and kidney index decreased obviously, while serum levels of BUN, SCr, and BUN increased visibly. Besides, rhein significantly reduced the content of proinflammatory factor TNF-*α* and antioxidative stress factor SOD and GSH-PX. Furthermore, 0.35 g/kg rhein caused swelling of renal tubules epithelial cells and small focal proliferation of lymphocytes [45]. Consequently, long-term and high-dose administration of rhein by oral administration can cause nephrotoxicity in mice, especially for the male animals. The potential toxic mechanism may be related to its inhibition of antioxidant pathways, triggering inflammation and inducing cell apoptosis.

## 4. Pharmacokinetics

At present, pharmacokinetic studies of rhein have been carried out in rats and beagles. Detailed pharmacokinetic parameters for these studies are shown in Table 3. Rhein is mostly administered orally due to its low solubility, but it is difficult to achieve stable and effective blood concentration. The pharmacokinetic behavior of rhein tended to be consistent between the two species. Moreover, the plasma concentration-time curve of oral administration rats was consistent with the two-compartment model, and the intravascular administration was consistent with the three-compartment model. Intravascular and oral administration in beagles was consistent with the three-compartment model. The absorption half-lives (*t*_1/2*α*_) and elimination half-lives (*t*_1/2*β*_) indicated that intravascular administration can be rapid excretion and metabolism in both rats and beagles compared with oral administration. In addition, the absolute bioavailability (F) of oral administration in rats and beagles was less than 50%, indicating low bioavailability of oral administration of rhein [102]. In addition, compared with the above two-compartment model [102] in rats, the half-life of oral administration was significantly prolonged in the noncompartmental pharmacokinetic parameter model PBPK (physiologically based pharmacokinetic). The cumulative excretion rate in bile and urine was 0.6% and 6.0% within 24 hours after oral administration of 70 mg/kg rhein. The renal clearance rate of rhubarb was 21.3 mL/hr/kg. The renal clearance rate of rhein was 21.3 ± 5.47 mL/hr/kg. It is speculated that rhein may be eliminated mainly through renal metabolism [103]. In short, comparing the pharmacokinetics of rhein in the body of two species (rats and beagle dogs) and two routes (oral and intravascular administration), the absorption of rhein in beagle dogs is slightly higher than in rats, and the absorption of intravascular route of administration is greater than that of oral administration.

## 5. Conclusion and Discussion

Rhein exists in many Chinese herbal medicines, such as Heshouwu and Dahuang, which are commonly used in clinic. Rhein has a variety of pharmacological effects, and corresponding mechanisms are quite complex and interrelated. It shows bidirectional actions in the liver and kidney. In addition, the application of rhein is limited to its structure. Modification of its phenolic hydroxyl or carboxyl group can improve the solubility and pharmacological targeting, which provides potential for further exploration of rhein derivatives.

### 5.1. Pharmacological Mechanism of Rhein

With the deepening of research on rhein, it has been fully proved that rhein has a variety of pharmacological activities, such as cardiocerebral protective effect, hepatoprotective effect, nephroprotective effect, anti-inflammatory effect, antitumor effect, antidiabetic effect, and antimicrobial effect. In addition, rhein also has antipancreatic fibrosis and antiallergic activity. These pharmacological effects indicate that the therapeutic properties of rhein are related to the reduction of oxidative stress, antifibrosis, anti-inflammation, regulation of cell apoptosis, inhibition of angiogenesis, regulation of energy metabolism, and so on. Further studies revealed that multiple signaling pathways are involved in different pharmacological effects. The molecular and cellular targets of rhein mentioned in this paper are shown in Figure 2. It is speculated that the cardiocerebral protective effect of rhein is mainly related to the inhibition of ROS overproduction and regulation of GSH/GSSG, Akt/GSK3 *β*/p38, MAPK, and NF-*κ*B signaling pathways. The regulation of these pathways will lead to the increase of oxidative stress-related factors ROS, SOD, CAT, and GSH, the decrease of GSSG and MDA, and phosphorylation of key proteins, such as JNK, Akt, ERK, p38, and p65. Besides, it can be inferred that the anti-inflammatory effect of rhein is related to NF-*κ*B, PPAR*γ*-NF-*κ*B-HDAC3 axis, and NALP3 inflammatory pathway. It is due to the inhibition of IKK *β* that rhein significantly enhances the secretion and phagocytosis of TNF-*α* in macrophages. Moreover, rhein also reduces the contents of IL-6 and IL-1*β*. In addition, the potential mechanisms of rhein to prevent tumors include inhibiting cell proliferation, migration and invasion, promoting cell apoptosis, inhibiting mitochondrial energy metabolism, and inhibiting angiogenesis. Akt, FOXO, p53, ER stress, and mitochondria-mediated pathway are involved in this effect. Furthermore, rhein-mediated diabetes treatment is associated with inhibition of PPAR*γ*, Drp1, 8-OHdG, and SREBP-1c expression and the increase of phosphorylation of START6, which plays an important role in glucose homeostasis, lipid synthesis, and adipocyte differentiation. Inhibition of the TGF-*β*1/Smad signaling pathway plays a vital role in antifibrosis effect of rhein, mainly including the downregulation of TGF-*β*1, *α*-SAM, FN, and TIMP-1, and the upregulation of MMP-13 and Smad7.

### 5.2. Bidirectional Regulation of Rhein on Liver and Kidney

The liver and kidney are both important organs in the human body for their significant functions. They are usually damaged by many factors in daily life, in which drug intake is one of the most common reasons [104]. Rhein, as the active ingredient of the Chinese herbal medicine Dahuang and Heshouwu, is usually taken by people as drug. In the pharmacological parts, we have concluded that it can improve liver/renal fibrosis by reducing the expression of smooth muscle actin (a-SMA) and fibronectin (FN) and inhibiting the expression of transforming growth factor-*β*1 (TGF-*β*1) and its type I receptor. In addition, rhein can also reduce drug-caused liver injury, such as APAP and MTX, by inhibiting oxidative stress [30, 31]. However, simultaneously, rhein alleviated hyperuricemia by promoting uric acid excretion [38] and the NF-*κ*B anti-inflammatory pathway [39] and also inhibited the demethylation of Klotho promoter, thus reversing the chronic kidney disease caused by Klotho loss [11]. Rhein is also effective on diabetic nephropathy by activating the Notch1-RBP-JK/Msx2 pathway [41]. In a nutshell, rhein has protective effect on the liver and kidney. However, studies also shows that rhein has potential hepatorenal toxicity, which is mainly related to the induction of oxidative stress, the destruction of liver drug enzyme activity, the triggering of inflammation, and the promotion of cell apoptosis. As shown in Table 2, when rhein exerts its protective effect on liver and kidney organs, the dosage of rhein varies from 20 to 150 mg/kg, and the administration time is usually less than 14 days mostly. However, oral administration of 0.175 g/kg or 0.35 g/kg rhein for 60 days could cause severe nephrotoxicity. Interestingly, rhein may achieve hepatic protection through the mitochondrial antioxidative stress pathway and can also cause hepatotoxicity by inducing hepatocyte apoptosis through the mitochondrial pathway. Coincidentally, rhein can alleviate various kidney diseases by anti-inflammation and antioxidant. On the other hand, rhein could also cause nephrotoxicity by promoting oxidative stress and inducing inflammatory reaction. This may be caused by the concentration and duration of rhein. These studies indicate that dosage and course of treatment are vital factors for rhein's bioactivities in the liver and kidney, protective or toxic. Besides, the mechanism and targets between protective effects and toxicity are different. Recently, studies have been shown that mitochondrial fission inhibiting by FUN14 domain-containing protein 1 (Fundc1) mitophagy [73] and promoting the retention of prohibition 2 (PHB2) [72] could maintain mitochondrial homeostasis, thereby resisting AKI. The nephroprotective effect of rhein on AKI in bidirectional regulation of rhein may also be achieved through these newly discovered targets, which maybe one of the future research directions.

### 5.3. Development of Derivatives

In the past research and clinical practice, it has been fully verified that rhein has a variety of pharmacological activities and is a natural product with good development prospects. To solve the problems of low solubility and low bioavailability of rhein, some rhein-related derivatives were synthesized by structural modification, and some of which processed better drug metabolism, solubility, or enhanced pharmacological effects and lower toxic and side effects [4, 6, 105]. The structural modification of rhein is mainly through the modification of the phenolic hydroxyl and carboxyl groups at positions 1, 3, and 9. Rhein generates esters and amide derivatives by substituting the carboxyl group at position 3 [106–109]. Among them, amide derivatives mostly enhance solubility, while ester derivatives can mainly enhance the pharmacological activity and targeting of drugs. For example, Li et al. found that HPDM-rhein can cross the blood-pancreatic barrier and specifically aggregate in the lungs, thereby improving acute lung damage caused by pancreatitis (AP) [109]. And rhein-phospholipid complex (RH-PLC) improves the solubility and skin permeability of rhein [110]. Multiple substitutions at other sites can enhance pharmacological activity, such as enhancing antitumor activity [111, 112] and antibacterial [113]. Even new pharmacological effects have been discovered, such as anti-Alzheimer's disease [114]. In brief, as shown in Table 4, the structural modification of rhein can increase the solubility of rhein and enhance its pharmacological action and targeting.

### 5.4. Clinical Significance of Rhein

Natural products are important sources of new drug development and application, and clarifying the relationships between the bioactivities of natural products and their structures are quite significant in the process of new drug development [115, 116]. Anthraquinone compounds [117] and phenolic compounds [118] are two important natural products, which may protect against or slow down inflammatory diseases, cancer, and cardiovascular diseases. Rhein belongs to anthraquinone compounds existing in a variety of commonly used traditional Chinese medicinals as pharmacological-related components, such as rhubarb. The 2020 edition of *Pharmacopoeia of the People's Republic of China* stipulates that the content of rhein in rhubarb is not less than 1.5%. Meanwhile, rhein is the main pharmacodynamic component and ingredient of quality control of some commonly used Chinese patent medicines, such as Dahuang Lidan Jiaonang (capsule) and Jiuzhi Dahuang Wan (pellet) [119]. Among them, Dahuang Lidan Jiaonang is used to treat acute cholecystitis [120] and nonalcoholic fatty liver [121], and Jiuzhi Dahuang Wan is applied to treat cirrhosis ascites [122]. Diacerein is a marketed drug for osteoarthritis, which has unique pharmacological actions and biological activities including anti-inflammatory, anticatabolic, and proanabolic properties on cartilage and synovial membrane clinically [123]. Furthermore, rhein is an active metabolite of diacerein [124, 125] and the only analyte detected in human plasma at present [126]. The study showed that the anti-inflammatory effect of diacerein on osteoarthritis must be owing to rhein's effect of inhibiting the level of IL-1 and stimulate cartilage regeneration in osteoarthritis [126], which indicates its great potential clinical values. Although rhein has a variety of pharmacological effects, its hepatorenal toxicity has attracted wide attention. Therefore, the hepatorenal toxicity dose of rhein is further summarized in this review in order to provide a safe dose range for the subsequent research and development of rhein as a candidate drug and also provide a basis for the safety risk evaluation and clinical use of traditional Chinese medicine containing rhein. Additionally, the pharmacokinetics of rhein are also summarized in order to provide the potential for development of rhein derivatives. Probably, modificating the structure of rhein to improve the bioavailability and increase drug targeting as well as lowering toxicity will be a hot topic in the future.

## 6. Future Prospects

Rhein has a variety of pharmacological activities, and clarifying its dose-time relationship will help determine the clinical therapeutic dose in the future. It is worth noting that the effects of rhein are different at different concentrations and time, and even toxic reactions occur under long-term or high-dose use. Therefore, the characteristics of hepatotoxicity and nephrotoxicity, and the toxic and effective doses for different diseases need to be further clarified, in order to provide basis for clinical adjustment dose. Then, in view of the characteristics of low bioavailability and solubility of rhein, further research on structural modification could be conducted in order to find derivatives with low toxicity and better efficacy, which is conducive to the development of new drugs. More importantly, recent research studies have new insights into the mechanism of tumors, AKI and MI, such as the role of mitochondrial homeostasis in myocardial I/R injury and AKI, and the importance of NF-*κ*B and ROS in the occurrence and development of cancer. Therefore, the mechanism of rhein in antitumor effect, nephroprotective effect, and cardioprotective effect should be further studied.

## Figures and Tables

**Table 1:** Pharmacological mechanism of rhein.

Effects	Pathways	Mechanism	References
Cardiocerebral protective effect	Antioxidant stress	Inhibiting the mRNA expression of Bid and caspase-3, -8, and -9; MDA↓, LDH↓, NO↑, NOS↑, SOD↑, and GSH-PX↑	[7]
	MAPK and NF-*κ*B signaling pathways	Inhibiting p-JNK/JUK, p-P38/P38, and p-P65/P65; ROS↓, GSH-PX↑, and CAT↑; IL-6, TNF-*α*, IL-1*β*, and IL-1*α*↓	[8]
	NADPH oxidase/ROS/ERK/MMP-9 signaling pathway	Inhibiting MMP-9 mRNA and protein; inhibiting GFAP, p-ERK, gp91^phox^, and ROS *in vitro*	[9]
	Antioxidant stress	SOD and CAT activity↑, GSH level and GSH/GSSG ratio↑, and MDA and GSSG↓	[10]
		NFS↓ and infarct size↓, MDA↓, SOD↑, GSH-PX↑, and CAT↑	[11]
	Akt/GSK3*β*/p38 signaling pathway	Promoting p-Akt and p-GSK3*β*, and inhibiting p-p38, but these effects were eliminated by PI3K/Akt inhibitor LY294002 or GSK3*β* siRNA	[12]

Anti-inflammatory effect	NF-*κ*B signaling pathway	Inhibiting NF-*κ*B and its downstream iNOS activation	[13]
	PPAR*γ*-NF-*κ*B-HDAC3 axis	Enhancing the binding of PPAR*γ*, NF-*κ*B, and HDAC3	[14]
	NLRP3	Inhibiting NLRP3	[15]
	LPS/TLR4/NF-*κ*B signaling pathway	Inhibiting TLR4 and phosphorylation of NF-*κ*B	[16]

Antitumor effect	PI3K/Akt signaling pathway	Inhibiting p-Akt	[17]
		Inhibiting PI3K, p-Akt, and p-ERK	[18]
	p53 signaling pathway	Increasing the release of ROS and the expression of ASK1 and p53	[19]
	Mitochondria-mediated signaling pathway	Promoting the expression of Cyt c and Apaf-1	[20]
		Reducing the ATP content, leading to the loss of MTP, promoting the release of Cyt c	[21]
	FOXO signaling pathway	Restraining p-Akt and stimulating FOXO transactivation activity	[22]
	MAPK signaling pathway	Promoting ERK, while inhibiting p-ERK	[23]
	Inhibiting AlkB repair enzyme	Inhibiting AlkB repair enzymes (AlkB, ALKBH2, and ALKBH3)	[24]

Antidiabetic effect	LXR-mediated negative energy balance, metabolic regulatory pathways	Inhibiting SREBP-1c and T-bet, enhancing the phosphorylation of STAT6 and GATA-3, changing Th1/Th2 reaction	[25]
	Antibacterial and anti-inflammatory pathways	Reversing the proinflammatory aggregation and microbiota changes in mouse large intestinal macrophages induced by the HF diet	[26]
	PPAR*γ* signaling pathway	Inhibiting PPAR*γ* and the expression of its target genes	[27]
	Drp1/NF-*κ*B/8-OHdG signaling pathway	Inhibiting mitochondrial Drp1, NF-*κ*B, and 8-OHdG	[28]

**Table 2:** Comparison of the liver and kidney activity of rhein and liver and kidney toxicity.

Effects	Dosage	Model/duration	Mechanism	References
Hepatoprotective effect	50 and 100 mg/kg rhein for 4 weeks	Rat liver fibrosis model i.p. 40% CCl_4_ 3 ml/kg twice a week for 3 weeks	Protein and mRNA expression of *α*-SMA, TIMP-1↓, and MMP-13↑	[29]
	10, 20, and 40 mg/kg of rhein	Liver toxicity i.g. 2.5 g/kg APAP	GPT, GOT, urea, CREA, and ROS levels↓, contents of NO, MDA, and GSH↓, and improved liver cell necrosis and steatosis	[30]
	20, 50, and 100 mg/kg of rhein for 7 days	Liver toxicity	Cell viability↑ and apoptosis rate↓; ALT and AST↓, mRNA and protein levels of Nrf2↑, Bcl-2↑, HO-1↑ and GCLC↑, and Bax↓; protein levels of NF-*κ*B↓, TNF-*α*↓, and caspase-3↓	[31]
		MTX (20 mg/kg, i.p.) at the 5-7th days		
	100 mg/kg rhein for 8 weeks	Nonalcoholic fatty liver disease (NAFLD) high-fat and high fructose for 8 weeks	ALT, AST, GLU, TC, TG, and MDA in the serum↓, SOD and GSH-PX↑. TLR4, MYD88, and Cyr61 in liver tissues↓	[32]

Nephroprotective activity	150 mg/kg rhein for 5 days	UUO-induced renal interstitial fibrosis in mice	a-SMA and the deposition of FN↓, TGF-*β* 1, and type I receptor↓	[33]
	120 mg/kg rhein (pretreatment for 1 day and posttreatment for 3 days	UUO-induced renal interstitial fibrosis in mice	Reversing aberrant expression of DNMT1/3a, maintaining the sKL and mKL levels, and inhibiting profibrotic protein expression	[34]
	120 mg/kg rhein	In a mouse model of adenine-induced chronic kidney disease (CKD)	Restoring the abnormal expression of E-cadherin, a-SMA, b-catenin, and phosphorylated Smad3. Correcting the hypermethylation induction of DNMT1/DNMT3a and Klotho promoter. The renal protective effect of rhein is basically eliminated by siRNA	[35]
	10 *μ*g/mL rhein	Adenine (Ade)-induced NRK-52E cells	Inhibited HBSS/LiCl-induced NRK-52E cell autophagy by regulating AMPK-dependent mTOR, ERK, and p38 MAPKs as well as Akt-independent signaling pathways	[36]
	50, 100, and 150 mg/kg rhein.	In a rat CKD model induced with 5/6 nephrectomy	Scr and BUN↓, ROS levels and the expression of NADPH oxidase subunits p47^phox^ and gp91^phox^↓, and alleviated renal interstitial injury and collagen fiber	[37]
		In H_2_O_2_-stimulated SIRT3 knockout HK-2 cells for 4 h	After stimulated with H2O2 in scrambled HK-2 cells, Foxo3*α*↑, ROS levels, and p47^phox^ and gp91^phox^ protein expression↓	
	75, 150, and 300 mg/kg rhein for 14 days	In a mice model of the uric acid nephropathy (UAN) induced by adenine (150 mg/kg) and ethambutol (250 mg/kg) for 14 days	Levels of Sur, Scr, and BUN and the activity of XOD↓, CCr↑, the serum uric acid level↓, and PGE2, THF-*α*, PEG2, IL-1*β*, and TGF-*β*1↓	[38]
	20, 40, and 80 mg/kg rhein for 7 days	The model of sepsis-induced acute kidney injury by injecting 10 mg/kg lipopolysaccharide (LPS) and cecal ligation and puncture (CLP) *in vivo*.	BUN, SCr, TNF-*α*, and IL-1*β*↓, attenuate circulating leukocyte infiltration and enhance phagocytic activity of macrophages partly impaired. Cell viability↑, MCP-1, and IL-8↓, and expression of phosphorylated NF-*κ*B p65, I*κ*B*α*, and IKK*β*↓	[39]
	100 mg/kg rhein for 8 weeks	High-fat diet for 8 weeks and i.p. streptozotocin (25 mg/kg) to induce obese diabetic nephropathy rat model	The content of MDA↓, the activity of SOD↑, and the excretion of urinary protein content↓, the blood sugar and body weight↓	[40]
	25, 50, 100, and 150 mg/kg rhein for 4 weeks	High-fat diet for 4 weeks and i.p. streptozotocin (35 mg/kg) to induce obese diabetic nephropathy rat model	The mRNA and protein expressions of Notch1-RBP-JK, Msx2 and Runx2 ↓, and *α*-SMA↑	[41]
			Notch1-RBP-JK/Msx2 pathway	

Hepatotoxicity	50, 75, and 100 µM rhein for 24h	HepaRG cells	The IC_50_ is 77.97 *μ*M. MMP↓, stopped the cells in S phase, and Fas, p53, Bax, caspase-3 and caspase-8, caspase-9, and other apoptosis-related proteins↑	[42]
	50 mM rhein	Rhein is incubated with 20 pmol/mL recombinant human CYPP subtypes (CYP1A1, CYP1A2, CYP1B1, CYP2A6, CYP2A13, CYP2D6, CYP2B6, CYP2C9, CYP2C19, CYP3A4, and CYP2E1)	CYP2C19 metabolizes rhein into an epoxy compound, which bound to intracellular mitochondria, resulting in excessive production of ROS, a significant increase in AST and LDH, respiratory chain dysfunction, and liver function is impaired, and glutathione depletion leads to cell death	[43]

Nephrotoxicity	25, 50, and 100 *μ*mol/L of rhein	HK-2 cells	LDH↑, Fas↑, FasL↑, FADD↑, mRNA expression of caspase-3 and caspase-8↑, Cyt c↑, caspase-8↓, cleaved caspase-3 and cleaved caspase-8↑	[44]
	0.175 and 0.35 g/kg rhein for 60 days	KM mice	TNF-*α* and SOD activity↓, caspase-3↑, GSH-PX↓, TGF-*β*1↑, and rhein significantly decreased the kidney index of male mice	[45]

**Table 3:** Pharmacokinetic studies of rhein.

Species	Route of administration	Dosage (mg/kg)	Pharmacokinetic parameters														References
			*t*_1/2ka_ (h)	*t*_1/2*α*_ (h)	*t*_1/2*β*_ (h)	*t*_1/2*γ*_ (h)	MRT (h)	*t*_max_ (h)	*C*_max_ (*μ*g/mL)	AUC_0-T_ (*μ*g·mL^−1^·h)	AUC_0-∞_ (*μ*g·mL^−1^·h)	CL (L/h)	CL/F (L·h^−1^·kg^−1^)	V/F (l/kg)	*V*_*d*_ (L)	*F* (%)	
Rats (SD)	Oral	35	0.0062 ± 0.0054	0.86 ± 0.26	3.22 ± 1.21		2.59 ± 0.96	0.42 ± 0.26	38 ± 13	70 ± 9			0.50 ± 0.07	2.26 ± 0.73		16.4	[102]
		70	0.11 ± 0.21	1.48 ± 0.77	3.68 ± 1.42		4.03 ± 0.46	0.50 ± 0.27	55 ± 12	164 ± 45			0.43 ± 0.12	2.24 ± 0.85		23.8	
		140	0.04 ± 0.03	3.53 ± 1.58	4.30 ± 1.55		4.48 ± 0.60	0.38 ± 0.14	67 ± 15	238 ± 43			0.60 ± 0.12	3.82 ± 1.66		19.4	
		35	3.2 ± 1.2					0.42 ± 0.26	37.9 ± 12.8		69.5 ± 9.1		0.503 ± 0.06				[103]
		70	3.6 ± 1.4					0.50 ± 0.27	50.6 ± 11.6		164.3 ± 44.7		0.426 ± 0.092				
		140	4.3 ± 1.5					0.40 ± 0.13	67.1 ± 14.6		237.8 ± 42.8		0.588 ± 0.091				
	i.v.	0.35		0.05 ± 0.04	0.52 ± 0.15	3.55 ± 1.35	1.26 ± 0.67				3.29 ± 1.00	0.071 ± 0.023			0.99 ± 0.69		[102]
		0.70		0.06 ± 0.03	0.47 ± 0.20	4.93 ± 1.50	2.36 ± 1.11				7.32 ± 2.72	0.10 ± 0.05			1.20 ± 0.63		
		1.40		0.12 ± 0.05	1.62 ± 0.80	4.47 ± 1.03	2.03 ± 0.53				14.08 ± 1.64	0.08 ± 0.01			2.49 ± 2.31		

Beagle	Oral	20	0.52 ± 0.52	1.88 ± 0.43	3.25 ± 0.85		4.29 ± 0.57	2.75 ± 1.84	9.71 ± 4.89	41.02 ± 17.64			0.56 ± 0.21	2.72 ± 1.29		49.7	
	i.v.	0.4		0.05 ± 0.07	0.44 ± 0.52	1.77 ± 0.93	0.95 ± 0.22		3.81 ± 0.65		1.50 ± 0.49						

**Table 4:** Derivative name, structural formula, and characteristic of rhein derivatives.

Name	Structural formula	Characteristic	Reference
Rhein-naproxen prodrug 7e	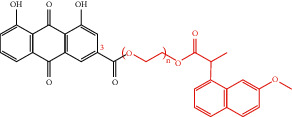	Improving drug metabolism	[106]
Rhein-hydroxyethyl hydroxamic acid derivative (SYSUP007)	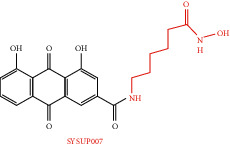		[107]
Rhein lysinate (RHL)	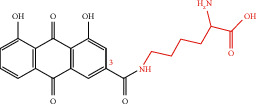		[108]
HPDM-rhein	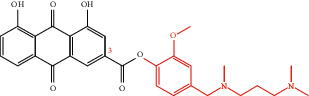	Improving anti-inflammatory effect	[109]
Rhein-phospholipid complex (RH-PLC)		Improving the solubility of rhein and skin permeability	[110]
Rhein derivative 4a	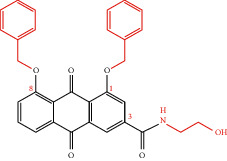	Improving antitumor effect	[111]
Rhein derivative 4F	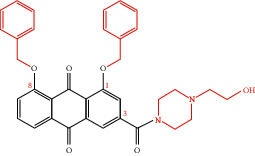		[112]
Metal complexes of rhein (rhein-Mn, rhein-Co, and rhein-Zn)	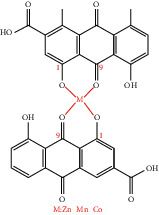	Improving antibacterial effect	[113]
Rhein-Huprine hybrid (±)-7e	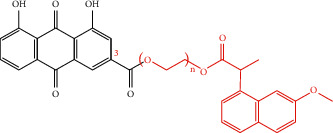	Anti-Alzheimer disease effect	[114]

## Data Availability

No data were used to support the findings of this study.
